# Hot Isostatically Pressed Nano 3 mol% Yttria Partially Stabilised Zirconia: Effect on Mechanical Properties

**DOI:** 10.3390/ma16010341

**Published:** 2022-12-29

**Authors:** Osamah Alsulimani, Julian Satterthwaite, Nick Silikas

**Affiliations:** 1 Dentistry, University of Manchester, Manchester M13 9PL, UK; 2 Faculty of Dentistry, King Abdulaziz University, Jeddah 21589, Saudi Arabia

**Keywords:** yttria stabilised zirconia, flexural strength, chewing simulation

## Abstract

**Objective:** To investigate the flexural strength of hot isostatically pressed nano 3 mol% yttria partially stabilised zirconia and conventionally sintered micro 3 mole% yttria partially stabilised zirconia. **Methods:** A total of 40 bar-shaped (2 mm × 4 mm × 16 mm) specimens were prepared from hot isostatically pressed nano 3 mol% yttria partially stabilised zirconia (CeramaZirc Nano HIP, Precision Ceramics) and conventionally sintered micro 3 mole% yttria partially stabilised zirconia (CeramaZirc, Precision Ceramics). Two groups were prepared for each material (n = 10), with one serving as ‘control’ and the other being cyclically loaded using a chewing simulator (7 kg; 250 k cycles): SEM imaging was also undertaken on selected specimens. Flexural strength until fracture was recorded (ISO 6872). Paired and unpaired *t*-tests were chosen to compare mean outcomes between the four groups (*p* < 0.05). **Results:** Significant statistical difference was only found between the means of control specimens. CeramaZirc Nano HIP had the highest mean value (1048.9 MPa), whilst the lowest was seen for CeramaZirc after loading (770 MPa). Values for both materials were higher without loading than after loading. Values after cyclical loading showed large SD values (276.2–331.8) in comparison to ‘control’ (66.5–100.3). SEM imaging after cyclical loading revealed a smoother and less destructed surface of CeramaZirc Nano HIP compared to CeramaZirc. **Significance:** HIP nano zirconia exhibited inferior strength, surface polishability and behaviour to loading. Therefore, further investigation on the behaviour of such materials should be conducted before recommending for clinical use.

## 1. Introduction

Zirconium dioxide (ZrO₂), known as zirconia, is a white crystalline oxide of zirconium’. Nevertheless, pure zirconium does not exist in nature, ‘but only in conjunction with silicate oxides (ZrO₂ × SiO₂) or as free oxide (ZrO₂)’ [1]. Moreover, it has an identical chemical and mechanical criterion to titanium [2]. In the late sixties, several trials were conducted to widen the use of zirconia as a biomaterial until 1969, when zirconia was first introduced to the medical field for orthopaedic purposes [3].

Zirconium was first introduced in dentistry as an endodontic post and implant abutment in the 1990s, and its use expanded afterwards [4]. That is attributed to its high physical criteria, bright colour, low thermal conductivity, resistance to corrosion, high tenacity, and high biocompatibility recommending its use in full-arch fixed and heavy occlusion cases [5]. The recently increased use of dental ceramics is reflected by the high claim for aesthetic restorations [1].

The use of zirconia and zirconia-reinforced restorations has increased over the last decade, especially with a drive for more aesthetically pleasing restorations [1]. However, zirconia is a polycrystalline ceramic and has almost no glassy matrix, and although this structure gives zirconia high strength, it impacts on the aesthetic aspect [6].

Zirconium has many crystalline forms: monoclinic, tetragonal, hexagonal and cubic polycrystals. Cubic and tetragonal polycrystals are able to be stabilised at room temperature with the addition of metal oxides during firing; the most used stabiliser is yttrium [7]. Addition of Yttria oxides enlarges the size of zirconia grain and reduces the coefficient of thermal expansion [8,9]. Restorations with yttria-stabilised tetragonal zirconia polycrystal (Y-TZP) have been shown to give good clinical outcomes [9]. Yttria oxide may be included at 3, 4, or 5 mole% [8]. 3 mole% Y-TZP has the highest mechanical stability with 85 to 90% tetragonal phase [8,10,11,12].

Continuous development of zirconium materials for dental use may revolve around nano-grain size with superior mechanical properties and upgraded translucency [8]: it has been proven that decreasing particles size influences optical and mechanical properties positively [13,14,15,16,17]. For instance, decreasing particle size influences slow T-M transformation, light transmission, decreases low thermal degradation, decreases stress induced-roughness, decreases modulus, and increases desirable crystalline structure stability [13,14,15,16,17]. Many techniques have been used to synthesise nano zirconium ceramics ranging from 5 to 200 nm grain size. However, there is a critical limit to the targeted size that should not be exceeded; otherwise, structural behaviour will be unfavourable. The limit is variable depending on multiple factors such as vicinity to phase transformation boundary [18,19]. Zirconia for use in dentistry is usually fabricated by cold pressing then being ground into blocks to be milled. During the last decade, zirconia has been introduced to the dental market with a lower opacity, allowing its use monolithically. However, fabricating milled monolithic zirconia restorations with good anatomical details is challenging; pressable zirconia ingots might be a solution as they can replicate the detailed anatomy done by the waxing-up process; however, this is not currently available in the dental market. Hot pressing was originally proposed post-sintering of zirconia to overcome processing defects [20,21]. Crack closure of surface defects has been illustrated by using heat treatment (annealing) [22,23], related to the progression of pore geometry during the heat treatment and reorganisation of polycrystals induced by the monoclinic to tetragonal phase transformation during the raise in temperature [22,23]. In the dental literature, annealing temperatures between 900 °C and 1000 °C have been successfully used on ground or sandblasted zirconia to reverse the monoclinic build-up phase back to the tetragonal phase [24]. Hot isostatic pressing (HIP) was proposed as an additional step after zirconia sintering to densify the particles using a high temperature of ~100–200 °C below the ideal sintering temperature of zirconia and isostatic gas pressure (argon) [21]. This process facilitates the transport of several mass procedures such as sliding of grain boundaries, diffusion-controlled creep and plastic deformation to better densification, pore shrinkage, and crack closure in zirconia without geometrical changes [25]. This HIP procedure after sintering is referred to as post-HIP or post-sinter HIP and has been presented to improve the fracture toughness of 3 mole% zirconia used for dental root posts [24].

CeramaZirc Nano HIP is a recently introduced non-dental pressed nano zirconia, which combines nano and pressing technology.

The aim of this study was to evaluate the strength pre- and post-loading of a recently introduced polycrystalline zirconia by comparing the flexural strength before and after chewing simulation between nano HIP 3 mol% yttria partially stabilised zirconia and conventionally sintered micro 3 mole% yttria partially stabilised zirconia to investigate the effect of hot pressing and nanotechnology on the behaviour of polycrystalline zirconia if pressable zirconia ingots to be used in dentistry. The Null hypotheses were: (i) there is no statistically significant difference between the flexural strength of conventional zirconia and HIP nano zirconia; (ii) there is no statistically significant difference between the pre- and post-loading flexural strength of conventional zirconia and HIP nano zirconia.

## 2. Materials and Methods

### 2.1. Materials

Two materials were selected: hot isostatically pressed nano 3 mol% yttria partially stabilised zirconia (CeramaZirc Nano HIP, Precision Ceramics, Birmingham, UK) and conventionally sintered micro 3 mole% yttria partially stabilised zirconia (CeramaZirc, Precision Ceramics, Birmingham, UK). Nano HIP has been recently developed, and at the time of the study was not yet commercially available. The mechanical properties according to the manufacturer are listed in Table 1.

### 2.2. Specimen Preparation

Both materials were fabricated and prepared by the manufacturer. A spray-dried powder was pressed to net shape in a die mould. This compact was then cold isostatically pressed at room temperature in a wet isostatic press (WIP) at high pressure, followed by sintering at high temperature (1550 °C) where the compact shrinks at approximately 20% and becomes ultra-hard; CeramaZirc was then cut from this. The rest of the tile (once cool), was then hot isostatically pressed (HIP) at 1450 °C to make CeramaZirc Nano HIP. Finally, both materials were diamond ground (126 µ grit) to form 40 bars (20 of each) of 2 mm thickness, 4 mm width and 16 mm length (ISO 6872). Specimens for each were divided into 2 groups: CeramaZirc Nano HIP (N1 and N2; n = 10); CeramaZirc (M1 and M2; n = 10). Specimens were dry stored before mechanical testing.

### 2.3. Flexural Strength Testing

A 3-point flexural strength test was conducted on M1 and N1 groups, using a Universal Testing Machine (Z020, Zwick/Roell, Leominster, UK) and TesXpert V11.02 software.

The machine was set as follows: 2 kN loading cell; 1000 mm softened upper switch; 200 mm lower softened switch; 1700 N upper force limit; 100 N lower force limit; 10 N pre-load; 1 mm/min loading arm speed (ISO 6872); 12 mm holding arms distance. The following equation was used to calculate flexural strength values:

(1) $$\sigma =\frac{3Pl}{2wb^{2}}$$

where:

*P* is the breaking load, in N;
*l* is the test span, in mm;
*w* is the width of the specimen, in mm;
*b* is the thickness of the specimen, in mm.

### 2.4. Chewing Simulation

**Figure 1:**
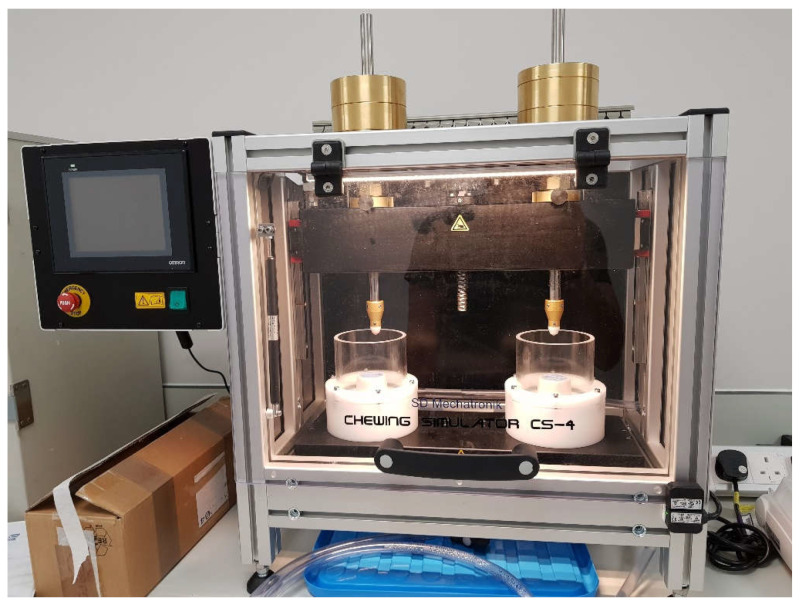
Chewing simulator CS-4.2.

**Figure 2:**
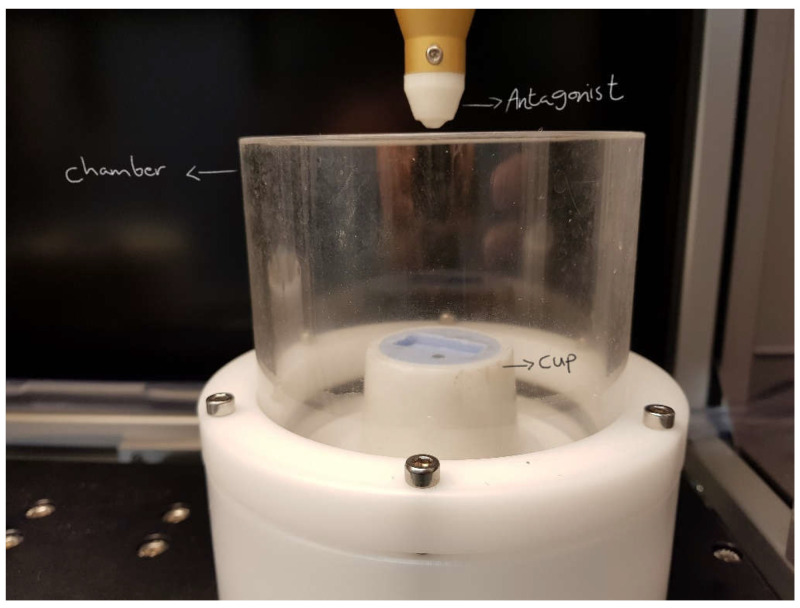
Chewing simulator components.

Cyclic loading was undertaken for M2 and N2 groups using a chewing simulator CS-4.2 (SD Mechatronik GmbH, Feldkirchen-Westerham, Germany) (Figure 1). The machine consists of 3 parts: dynamic antagonist (Steatite ceramic ball, Ø6 mm), specimen cup and specimen chamber (Figure 2).

The specimen cup was filled with silicone (Provil novo, Kulzer GmbH, Hanau, Germany) to form a mould and to simulate the cushioning effect of the periodontal ligament. The mould was formed by filling the cup with a silicon mix. Before the silicon was set, a rectangular acrylic piece (25 mm × 13 mm × 3 mm) was seated into the silicone with its surface flush with the silicon surface. Once the silicon was set, the acrylic piece then removed leaving its negative imprint, which was filled later with a clear orthodontic acrylic powder and liquid (Orthoresin, DeguDent GmbH, Hanau-Wolfgang, Germany), which once it was set, it formed a slab.

Afterwards, the acrylic slab was heat-cured under 2 bar pressure at 45 °C for 8 min (Palamat elite, Kulzer GmbH, Hanau, Germany). The clear acrylic slab was finished and polished using an acrylic grinder (Bracon Dental LTD, Heathfield, UK) and a rubber polisher (Identoflex Lab-Mini Pre polishers, Kerr Dental, Bioggio, Switzerland) underwater, then sandblasted along with the specimens in groups M2 and N2 with 110µm Al₂O₃ particles (Korax 110, BEGO Bremer Goldschlagerei, Bremen, Germany) at a pressure of 2 bar (Basic Classic sandblaster, Renfert GmbH, Hilzingen, Germany), at a distance of ~10 mm, at 90°, for 10 s using a linear motion. A separate acrylic slab was made for each test specimen.

The zirconia specimens for each group were positioned in the middle and adhesively cemented onto the acrylic slabs using Panavia F 2.0 (Kuraray Medical Inc., Tokyo, Japan) following the manufacturer instructions for cementing zirconia restorations, and light-cured for 20 s from each side (SmartLite Focus, DentSply Sirona, Germany). Once the specimens were adhesively cemented onto the acrylic slabs, slabs were seated back to their negative imprints in the mould cups. The chewing simulator machine has two chambers; each chamber can house a single cup as shown in Figure 2, hence, two specimens can be tested at a time. The specimen chamber was filled with distilled water. The chewing machine was set as follows: vertical volume Z 2.0 mm; lateral movement X 0.7 mm; vertical speed 55 mm/s; lateral speed 30 mm/s; frequency of 1.8 Hz; 7 kg (68.6 N) loading weight; 250 k cycles. The weights were mounted over the extension of the antagonist arm. The machine took ~48 h to complete 250 k cycles for every set.

After chewing simulation, to prepare the specimens for SEM scanning and post-cyclic loading mechanical testing, they were detached from the acrylic slabs using an 11 mm hollow acrylic grinder (Bracon Dental LTD, Heathfield, UK) to remove the acrylic slab around the specimen and a grey torpedo rubber pre-polisher to remove the remaining thin cement layer (Identoflex Lab-Mini Pre polishers, Kerr Dental, Bioggio, Switzerland).

### 2.5. Scanning Electron Microscopy

**Figure 3:**
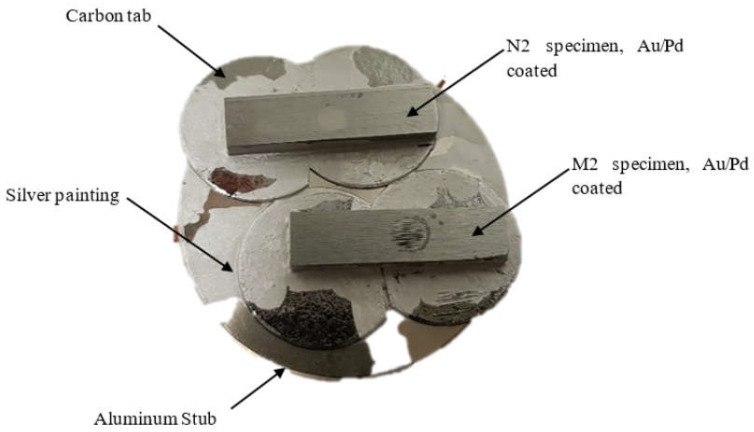
Specimens preparation for SEM imaging.

After cyclic loading, one sample of M2 and N2 were placed on carbon tabs and mounted on aluminium stubs (Figure 3). 10 nm Au/Pd coating was applied at 40 mA current and 16.40 g/m^3^ density for 120 s at room temperature (Q150T ES, Quorum Technologies Ltd., East Sussex, UK), followed by silver painting around the specimens to reduce charging. The specimens were cleaned then with 50 psi air pressure. The specimens were scanned with SEM using secondary electrons (SE) at 5 eV (MIRA 3 FEG, TESCAN, Brno–Kohoutovice Czech Republic) at different magnification (40 kx, 7.62 kx, 2 kx, 134 x, 60 x).

### 2.6. Statistical Analysis

**Figure 4:**
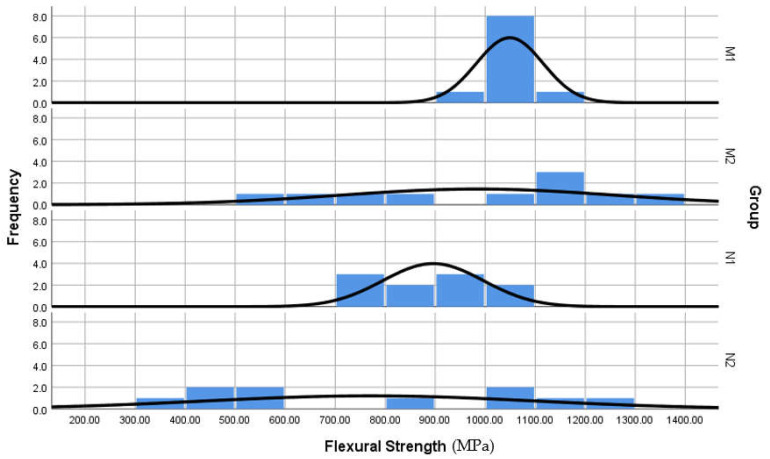
Frequency histogram polygon graph of the flexural strength stacked by group, comparing the pattern of values distribution between different groups.

**Figure 5:**
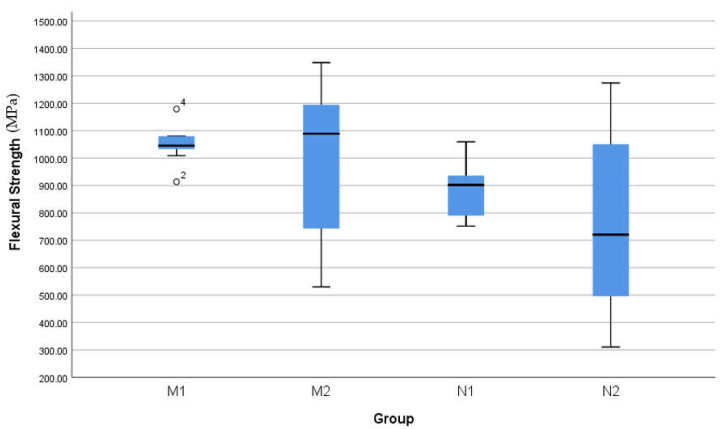
Box and whisker plots of the flexural strength, comparing the range of values spread between groups. Note the outliers in M1, but still considered high values.

Histogram polygon graphs showed normal distribution of the outcomes through all experimental groups; M2 and N2 groups were skewed by a negligible degree (Figure 4). Thereby, parametric paired and unpaired *t*-tests were chosen to compare mean outcomes between the groups (*p* < 0.05). Normality and equal variance were verified (Levene’s test > 0.05). Box and whisker plots used to compare the range of values spread between groups and to spot outliers (Figure 5). Statistical analysis was performed using IBM SPSS version 22 (SPSS Inc., Chicago, IL, USA).

## 3. Results

### 3.1. Flexural Strength

Values for each group are listed in Table 2. M1 showed the highest mean (1048.9 MPa) whilst N2 mean was the lowest (770 MPa). All means lie within the 95% confidence interval. Standard deviation values were noticeable larger after cyclic loading than before (Table 2). The spread of values between groups before and after cyclic loading is shown in Figure 4 and Figure 5. Two outliers were found within M1, but they were mild, thus, included in statistics.

There was no significant difference between the means of M1-M2 nor between means of N1-N2 (Table 3). There was a significant difference between the means of M1-N1 (*p* < 0.05), but not between M2-N2 groups (Table 3).

### 3.2. SEM Imaging

**Figure 6:**
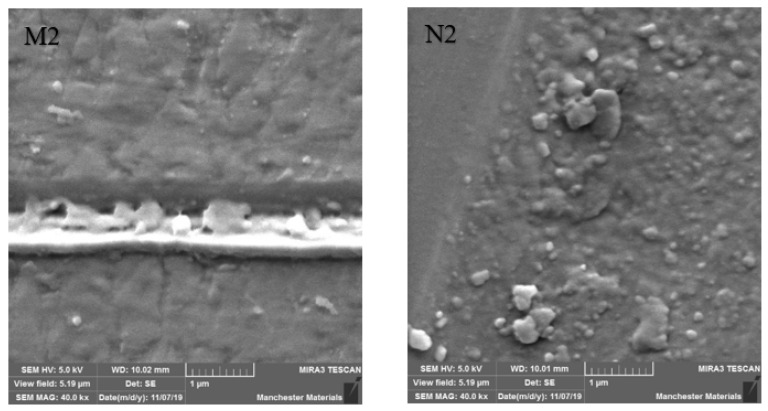
SEM imaging at 40 kx magnification at the point of impact. Note that the width of destruction and amount of particle debris are larger in N2 compared to M2.

**Figure 7:**
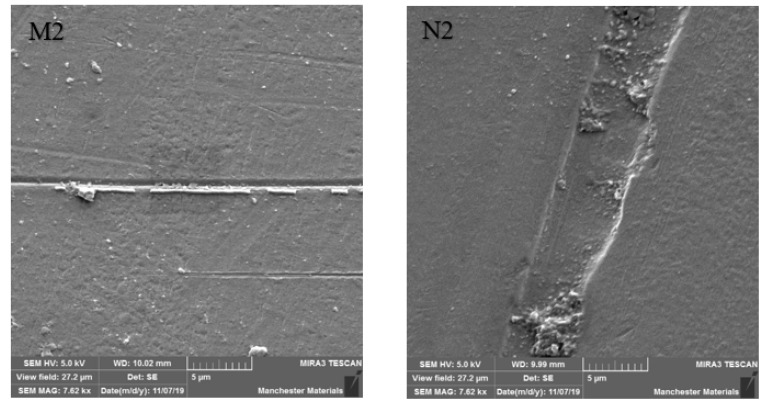
SEM imaging at 7.62 kx magnification at the point of impact. Note that the width of destruction and amount of particle debris are larger in N2 compared to M2.

**Figure 8:**
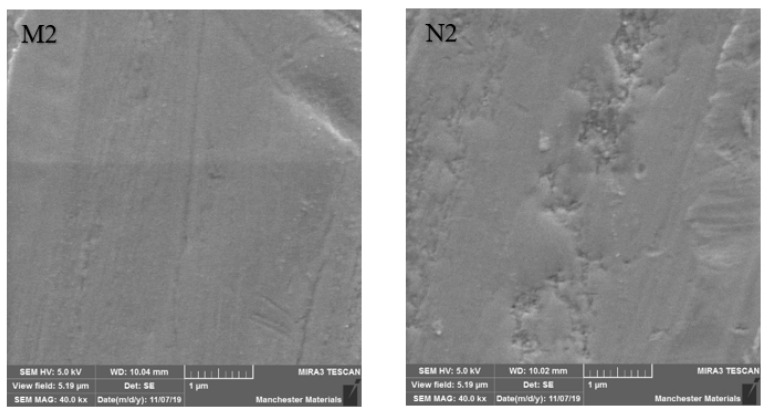
SEM imaging at 40 kx magnification at a point away from impact. Note smoother M2 surface compared to N2.

**Figure 9:**
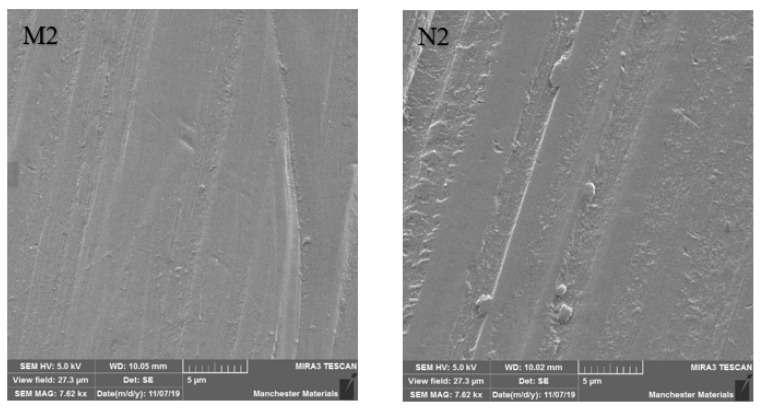
SEM imaging at 7.62 kx magnification at a point away from impact. Note smoother M2 surface compared to N2.

**Figure 10:**
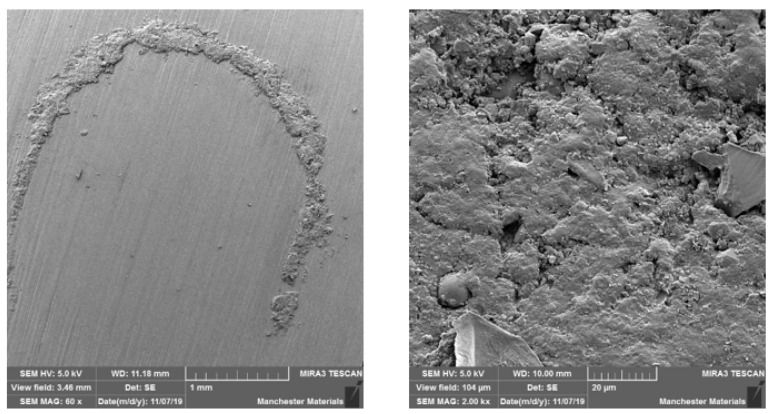
SEM imaging of N2 at 60 x (left) and 2 kx (right) magnification, showing exaggerated loading-induced particle debris. Note in the left image, particle debris rim surrounding the point of antagonist loading.

SEM imaging highlighted surface differences with M2 showing better surface texture, depth of wear and particle chipping in comparison to N2, which was more noticeably damaged. Surfaces at the point of impact exhibited grooving, which was broader and more profound in N2 (Figure 6 and Figure 7). Figure 8 and Figure 9 show a smoother surface for M2 at points away from impact. Numerous particle debris were distinct for N2, especially at the point of impact (Figure 10).

## 4. Discussion

The first null hypothesis was rejected, as conventional zirconia exhibited a high and consistent statistically significant pre-loading strength compared to HIP nano zirconia (*p* < 0.05). The second null hypotheses was accepted as there was no significant difference between paired groups for pre- and post-loading flexural strength; however the post-load behaviour was notably different with a much broader range of failure.

Cyclic loading affected both zirconia materials with flexural strength values widely spread around the mean (conventional, SD = 276.2; nano, SD = 331.8) as illustrated in Table 2 & Figure 6. The spontaneous and continuous transformation from a tetragonal to monoclinic phase results in wear-prone restorations, and is known as low thermal degradation (LTD): this can be induced by long-term surface exposure to water, vapour, body fluid or steam sterilisation [26]. Bigger particles sizes have been shown to give worse [14], with small particles size undergoing slow phase transformation, and consequently slower LTD [16]. These findings conform to the notion of favouring a non-hot-pressed nano zirconia model. An elicited hypothesis is that since chewing simulators apply continuous constant loads for an extended period, it might have induced a cascade of tetragonal to monoclinic phase transformation similar to that of LTD, and hence explain the wide SD range post-loading. It can be argued that this correlates clinically to the effect of bruxism, and that zirconia restorations are better avoided in bruxists, especially those with group function excursive guidance, due to the potential for unpredictable behaviour of the material under such circumstances. Additionally, volume expansion coupled with tetragonal to monoclinic phase transformation can lead to unpredictable frequent stresses and strains between particle boundaries of the crystalline structure, which may lead to fracture of the particles [27]. Very few in vivo trials have investigated the short-term service of zirconia restorations in bruxists. In 2019, Levartovsky et al. reported excellent short-term survival of anterior veneered and posterior monolithic 3 mole% YTZ crowns and 3-unit bridges [28]. At the same year, Contradictingly, Koenig et al. stated a weakness in the performance with sub-optimal short-term survival rate and 80% catastrophic failures in a bruxism group after observation of posterior monolithic 3 mole% YTZ crowns and 3-unit bridges [29]. An in vitro study to simulate the effect of bruxism found the majority of specimens fractured before 50,000 cycles [30]; 1.2 million cycles equivalent to 5 years in vivo [31]. Thereby, authors advise using zirconia with caution in such cases and not to be misguided by advertisements until medium and long-term service is investigated, due to high risk of core fracture [32].

HIP process (‘post-HIP’ or ‘post-sinter HIP’) has been claimed to improve mechanical properties due to overcoming/reducing manufacturing-related defects [24]. In the dental literature, pressing temperature between 900 °C and 1000 °C has been successfully used on ground or sandblasted zirconia to reverse the build-up of monoclinic phase back to tetragonal [24]. Of note is that those experiments considered other types of ceramics, such as zirconia-toughened alumina, rather than polycrystalline zirconia [20]. The present study, however, showed no improvements for the 3 mole% Y-TZP after HIP either mechanically or structurally. Our results conform to a previous study assessing the effect of hot isostatic pressing on the mechanical properties and defect closure of zirconia after sintering that found post-hot isostatic pressing compromised the strength and could not close any of the existing critical processing flaws as revealed by fractographic analysis after strength testing; hence, the monoclinic to tetragonal phase transformation did not lead to defect healing [21]. The authors hypothesised that the degree of grinding before HIP resulted in a high amount of pseudo-cubic transformation, which could not be reversed back to tetragonal form post-sinter HIP. Interestingly, they also observed that post-sinter HIP created defects larger than that created by grinding by 120 µm diamond particles, and this may also explain the inferior strength and surface behaviour of the HIP nano zirconia seen in our study. This is also supported by the sharp drop in flexural strength seen after exposing hot-pressed yttrium-stabilised tetragonal zirconia polycrystals to air ageing at temperatures between 800 °C and 1200 °C [33]. The HIP specimens in our study were pressed at 1450 °C which is high in comparison to other reported values and this may also have further contributed to the adverse structural properties; optimal pressing temperature requires further evaluation.

It has been shown that polished zirconia restorations lead to less wear of antagonistic teeth than feldspathic porcelain, lithium disilicate and nickel-chromium restorations [9,34,35]. With regard to surface texture and the potential for antagonist wear, the influence of composition and material degradation (ultimately leading to fracture) was also highlighted by the SEM imaging with the depth and width of impact being exaggerated for HIP nano zirconia, as seen in Figure 6, Figure 7 and Figure 10. Conventional zirconia exhibited smoother surfaces (Figure 8 and Figure 9) with less particle debris at the point of impact (Figure 6 and Figure 7), indicating higher polishability. This also limits the clinical applicability of HIP nano zirconia.

Although HIP nano zirconia exhibited lower strength than conventional 3 mole% zirconia, its strength is equivalent to conventional 4 mole% zirconia, which is suggested as an option for the fabrication of up to 3-unit posterior fixed bridges [36,37,38,39,40,41,42,43]. Despite this mechanical equivalence, there is little rationale for its use unless it could exhibit significantly better aesthetics monolithically or superior bond strength. Further investigations of HIP nano zirconia properties and behaviour are required before it can be recommended for clinical use in dentistry [44,45,46]. The crystalline structure, structural integrity, and influence of pressing (including temperature) and nano-technology of HIP nano zirconia deserve further study to investigate its inferior strength and behaviour.

## 5. Conclusions

Within the limitations of this study, the following was concluded:

1. Conventional micro zirconia exhibited high flexural strength.
2. Conventional micro zirconia has shown less particle debris around the impact point after loading, indicating higher structural integrity and a smoother surface, suggesting higher polishability.
3. Nano HIP zirconia and conventional micro zirconia were affected significantly after cyclic loading, and are not recommended for use in bruxists.

## Figures and Tables

**Table 1:** -Commercial names and properties of the tested materials.

	CeramaZirc Nano HIP	CeramaZirc
Flexural strength [MPa]	1400	850
Compressive strength [MPa]	2100	
Fracture toughness Kıᴄ [MPa/m²]	8	
Thermal expansion coefficient [×10⁻⁶/°C]	10	

**Table 2:** Descriptive Statistics.

	N	Mean (MPa)	Std. Deviation	Std. Error
M1 Group	10	1048.9	66.5	21
M2 Group	10	985	276.2	87.3
N1 Group	10	895.8	100.3	31.7
N2 Group	10	770	331.8	104.9
Total	40	924.9	240	37.9

**Table 3:** Summary of analysis.

		Sig. (2-Tailed)
Paired t	M1-M2	0.506
Paired t	N1-N2	0.286
Unpaired t	M1-N1	0.001 *
Unpaired t	M2-N2	0.133

* *p* > 0.05

## Data Availability

Not applicable.
